# Surgical Margin Analysis in Osteosarcoma: Impact on Survival and Local Control

**DOI:** 10.3390/cancers17152581

**Published:** 2025-08-06

**Authors:** Sebastian Breden, Simone Beischl, Florian Hinterwimmer, Sarah Consalvo, Carolin Knebel, Rüdiger von Eisenhart-Rothe, Rainer Burgkart, Ulrich Lenze

**Affiliations:** Department of Orthopaedics and Sports Orthopaedics, TUM Klinkum Rechts der Isar, Ismaninger Str. 22, 81675 Munich, Germany

**Keywords:** bone cancer, sarcoma, musculoskeletal tumors, sarcoma surgery, orthopedic oncology

## Abstract

Background/Objectives: The quality of surgical margins has been shown to be a prognostic factor in many sarcoma entities, yet its role in osteosarcoma remains controversial. While previous studies have shown that the outcome was not related to the margin width in bone, the impact of the extraosseous margin width (margin at the soft tissue invasion)—which needs to be close sometimes due to neurovascular structures—needs to be assessed. This study aims to evaluate the influence of soft tissue surgical margins on local recurrence and overall survival in patients with high-grade osteosarcoma. Methods: We conducted a retrospective, single-center study including 75 patients treated for high-grade osteosarcoma. All patients underwent standardized neoadjuvant chemotherapy followed by complete surgical resection. Patients were stratified into three groups based on the histological margin width of the extraosseous parts: group 1 (<1 mm), group 2 (1–5 mm), and group 3 (≥5 mm). Primary endpoints were local recurrence and overall survival (OS), analyzed using Kaplan–Meier estimates, log-rank tests, and Cox regression. Results: Local recurrence occurred in seven patients (9%). Although the overall comparison between the three groups was not statistically significant (*p* = 0.074), a subgroup analysis revealed a significantly higher recurrence rate in patients with margins < 1 mm compared to those with wider margins (*p* = 0.024). No significant differences in overall survival (OS) were observed between the groups (*p* = 0.896). Tumor location, metastatic status, and UICC stage were significant predictors for both endpoints in univariate analysis. However, none of these association were confirmed in multivariate analyses. Conclusions: Very close surgical margins (<1 mm) may increase the risk of local recurrence in high-grade osteosarcoma; however, they do not appear to affect overall survival.

## Simple Summary

Osteosarcoma (OS) is a rare but aggressive bone cancer that requires surgical resection to remove the tumor completely. In some cases, the tumor is located in close proximity to important nerves and blood vessels, making it difficult to remove with wide safety margins. This study looked at whether the size of the margin left around the tumor during surgery affects the local recurrence rate and overall survival. By reviewing 75 patient cases, we found that when the margin was extremely small (less than 1 mm), OS was more likely to relapse in the same area. However, this did not seem to affect how long patients lived. These results suggest that very close surgical margins might require more attention or follow-up treatment, but may still be acceptable with respect to survival rates.

## 1. Introduction

Osteosarcoma (OS) is the most common primary malignant bone tumor, with an annual incidence of 0.2–0.3 cases per 100,000 individuals [1,2,3]. It primarily affects the metaphyseal regions of long bones, especially around the knee [4,5], and is characterized by the production of osteoid by malignant osteoblasts [6]. While advances in chemotherapy and surgical techniques have improved survival, outcomes remain suboptimal in high-risk cases—particularly in the presence of metastases, poor histologic response to chemotherapy, or inadequate surgical margins [7,8].

Historically, radical surgery, often including amputation, was considered the standard of care [9,10]. However, limb-sparing surgery (LSS), in combination with effective chemotherapy protocols, has led to improved functional and oncological outcomes [11,12].

Complete resection remains a prerequisite for achieving local control and long-term survival [13,14,15]. However, the significance of the width of surgical margins—defined as the minimum distance between the tumor and surrounding healthy tissue—continues to be debated [16,17].

As long as the resection is complete, almost all studies suggest no prognostic difference between close or wide margins in sarcoma surgery in general [18,19] and in osteosarcoma specifically [8,14,20,21]. Nonetheless, in cases of very close margins (<1 mm), especially when accompanied by a poor response to neoadjuvant chemotherapy, adjuvant radiotherapy is suggested by some authors [22,23].

Very close margins may be inevitable when tumors invade into the soft tissue and are located adjacent to critical neurovascular structures, limiting the extent of surgical resection. A limited number of studies concerning this question have been published. Loh et al. and Andreou et al. assessed bone resection after osteosarcoma resection and found no association between margin width and survival [8,20]. Bertrand et al., in contrast, evaluated margins in soft tissue invasion rather than in the bone, but could not find any correlation either [14]. In 2016, He et al. published a meta-analysis reporting a higher recurrence rate with narrower margins [13]. However, in this review, the primary sources used widely different methodologies and were very difficult to compare. Importantly, none of these studies specifically analyzed outcomes in patients with margins < 1 mm.

Close but adequate margins are increasingly being discussed in the context of further local treatments such as radiotherapy [22,23]. The aim of this study was to evaluate the relationship between the surgical margin width at the soft tissue invasion with regard to overall survival and local recurrence rate in osteosarcoma patients.

## 2. Materials and Methods

This retrospective, monocentric study was conducted at our specialized musculoskeletal tumor center. The study included patients treated for osteosarcoma between 2004 and 2024. Patients were selected through a review of our institutional database. All patients diagnosed with osteosarcoma were screened and their medical records were analyzed. The data collected included demographic information, tumor characteristics (e.g., size, subentity, grading, location, and presence of metastases), and histopathological reports following surgical resection (response to neoadjuvant chemotherapy and surgical margin width at the soft tissue invasion). Additionally, we reviewed each patient’s medical history, including (neo) adjuvant treatments, follow-up surgeries, local recurrence, and the date of death or the last presentation at our clinic/elsewhere.

The width of soft tissue surgical margins was obtained from histopathological reports. For statistical analysis, the cohort was stratified into three groups:

Group 1: Margin < 1 mm.

Group 2: Margin 1–5 mm.

Group 3: Margin > 5 mm.

The tumor response to neoadjuvant chemotherapy was categorized according to the system described by Salzer-Kuntschik et al. [24], where Grades 1–3 (with <10% vital tumor remnants) were classified as a good response, and Grades 4–6 (with >10% vital tumor remnants) were considered as a poor response.

Only patients with histologically confirmed high-grade osteosarcoma and complete surgical resections following neoadjuvant chemotherapy were included in the study. Low-grade tumors, osteosarcomas of the skull, high-grade tumors without neoadjuvant chemotherapy, tumors without soft tissue invasion, and patients with pathological fractures as well as patients with previous operations or who have been treated at other institutions were excluded.

The primary endpoints of the study were local recurrence, defined as histologically confirmed tumor growth at the surgical site, and overall survival (OS). Univariate and multivariate survival analyses were performed with all gathered data.

Data was analyzed using SPSS version 29 (SPSS Inc., Chicago, IL, USA). Descriptive statistics were used to characterize the patient population. Differences between the cohorts for all variables were assessed using the log-rank or ANOVA tests. Kaplan–Meier curves were generated for overall survival (OS) and recurrence-free survival. Multivariate analysis using Cox regression was performed to identify risk factors for these endpoints, respectively. A *p*-value of less than 0.05 was considered statistically significant.

The study was approved by the Ethics Committee of the School of Health and Medicine at the Technical University of Munich (Approval No. 48/20S; 21 March 2021). Given the retrospective nature of the study, informed consent was waived as all data were anonymized.

## 3. Results

### 3.1. Patient Demographics

Between 2004 and 2024, a total of 221 patients with osteosarcoma were treated at our tertiary tumor center, of which 156 underwent primary surgical treatment at our institution. After excluding cases with low-grade tumors, previous operations, incomplete resections, pathological fractures, or insufficient data, 75 patients could be included in this study. The patient cohort consisted of 40 males (53%) and 35 females (47%), with a mean age of 22 years (range: 6–69 years) at the time of resection. The majority of tumors occurred in the lower extremities, with 38 tumors (51%) located in the femur and 18 (24%) in the tibia. Demographic data are shown in Table 1. No significant differences in demographics were observed when tested between each of the groups.

### 3.2. Tumor Characteristics

The majority of patients presented with conventional osteosarcomas, including 43 (57%) osteoblastic, 13 (18%) chondroblastic, and 9 (13%) mixed-type tumors. Maximum tumor sizes ranged from 25 mm to 320 mm, with an average size of 104 mm. Tumor grading was assessed based on biopsy results prior to neoadjuvant chemotherapy, following the FNCLCC classification [12]. Of all patients, 7 (9%) had an intermediate-grade (G2) osteosarcoma, and 68 (91%) had a high-grade (G3) osteosarcoma. At the time of resection, the TNM classification according to UICC was documented [25]. Stage 1 cancer was present in 25 cases (33%), while stage 2 was the most common, affecting 45 patients (60%). Stages 3 (3; 4%) and 4 (2; 3%) were less frequently seen. Tumor characteristics are presented in Table 2. In our patients, 28 (38%) had metastases, 14 (19%) synchronous metastases at the time of diagnosis, and 14 (19%) developed metachronous metastases during the course of treatment.

### 3.3. Treatment

Neoadjuvant and adjuvant chemotherapy was performed according to the contemporary protocol (COSS, EURAMOS or Euro B.O.S.S). No additional treatments were administered. Surgical resections were either wide or radical, with clear margins confirmed upon histopathological examination in all cases. At the soft tissue invasion, very close margins (<1 mm) were found in 8 cases (10%), close margins (1 mm up to 5 mm) were present in 41 cases (55%), and wide margins (≥5 mm) in 26 cases (35%).

In all cases, bone resection margins were histologically negative. Notably, the minimal bone margins were wider than the corresponding soft tissue margins in groups 1 and 2, and they were at least 2 cm in group 3.

According to Salzer-Kuntschik et al., the effect of neoadjuvant chemotherapy was graded as Grade 1 in 2 patients (3%), Grade 2 in 13 patients (17%), and Grade 3 in 16 patients (21%). Thus, a good response (≤10% viable tumor cells) was seen in 31 patients (41%), while the majority of tumors (44 patients; 59%) showed poor response to chemotherapy (poor responders with >10% viable tumor cells). Among the poor responders, 25 patients (33%) were classified as Grade 4, 16 (21%) as Grade 5, and 3 (5%) as Grade 6.

The groups according to the resection widths are shown in Table 1. Histopathological findings after resection are presented in Table 3.

In 7 patients (9%), a biological reconstruction was performed, and in 46 patients (57%) an endoprosthetic reconstruction was performed. Limb-sparing surgery without reconstruction was performed in 8 cases (11%), and 14 patients (19%) required amputation. These findings are summarized in Table 3.

### 3.4. Recurrence

Overall, seven patients (9%) experienced local recurrence, all of whom were treated with secondary wide or radical resections. In total, three local recurrences were found in patients with very close margins (group 1) after a mean of 31 months, three in patients with close margins (group 2) after a mean of 27 months, and one in a patient with wide margins (group 3) after 8 months.

**Figure 1:**
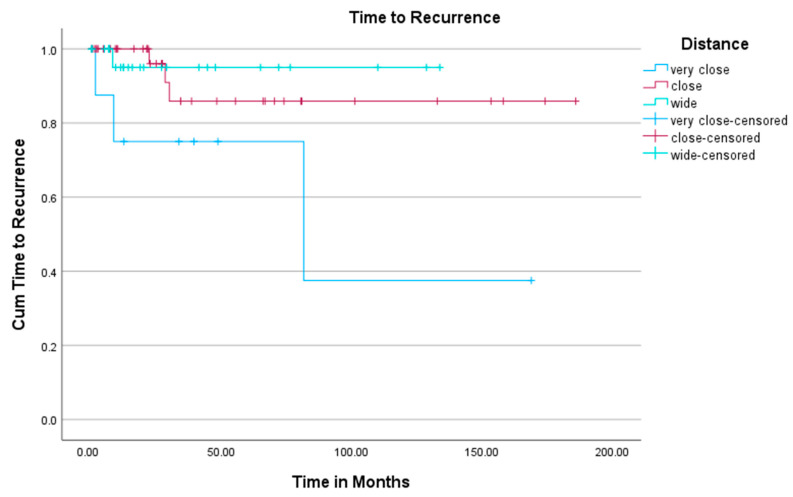
Kaplan–Meier plot visualizing the time to local recurrence between each of the study groups.

The overall comparison of the three groups using the log-rank test was not statistically significant (*p* = 0.074; Figure 1). However, a combined comparison of group 1 versus the pooled groups 2 and 3 showed a significant difference (*p* = 0.024), whereas the comparison of group 3 versus the pooled groups 1 and 2 was not statistically significant (*p* = 0.703).

### 3.5. Survival

**Figure 2:**
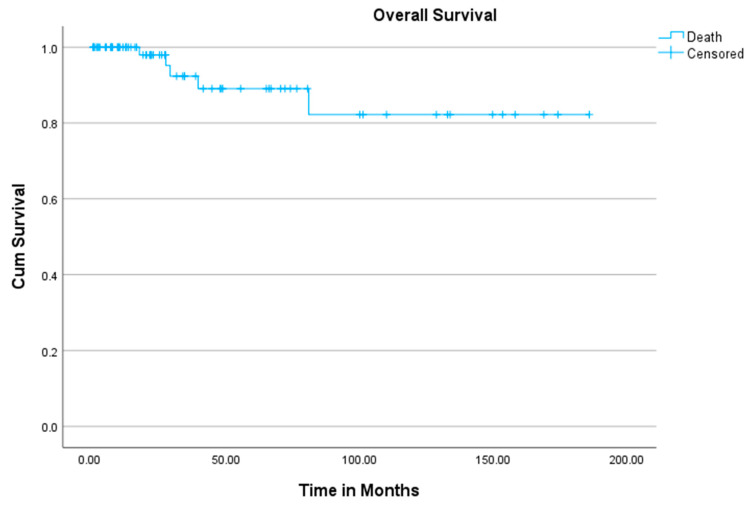
Overall survival.

The mean follow-up time for our cohort was 46 (1 to 186) months. At the end of the follow-up period, 70 patients (93%) were alive and 5 had deceased (7%) due to tumor-related reasons. The mean overall survival (OS) was 162 months (95% CI: 141–182), with a 5-year survival rate of 89% and a 10-year survival rate of 82% (Figure 2).

The mean survival time of deceased patients (5) was 46 months. The patient in group 1 died after 27 months, the one in group 3 after 81 months, and the three deaths in group 2 occurred after a mean of 54 months. The overall comparison of survival curves using the log-rank test did not reveal a statistically significant difference among the three groups (*p* = 0.896). Similarly, none of the individual comparisons showed significant differences.

### 3.6. Univariate Analyses

Univariate analyses were performed for each nominal and ordinal dataset gathered for this study concerning LR and OS.

Three factors showed significant differences in local tumor recurrence using univariate analyses. Patients that presented with or developed distant metastases during the follow-up had a significantly higher risk of recurring tumors than patients with localized diseases (*p* = 0.003). The UICC stage was another significant predictor of recurring tumors (*p* = 0.024) with a significantly higher risk in T3 and T4 cases compared to stage 1. The tumor location was a highly significant factor, with tumors of the pelvis resulting in significantly more recurrences than those of the extremities (*p* < 0.001).

In terms of overall survival, the univariate analyses revealed the same three significant factors. Patients with localized diseases survived significantly longer than those developing metastases (*p* = 0.002). The UICC stage was another significant factor (*p* < 0.001), with stage 4 patients dying significantly earlier than stage 1 or 2 patients. Lastly, the location was another significant factor (*p* = 0.001), with pelvic tumors resulting in worse survival rates than tumors of the limbs (Table 4).

### 3.7. Multivariate Analysis

Two separate multivariate analyses using Cox regression were conducted to identify factors associated with local recurrence and overall survival (OS). The first model included all three margin width groups as defined previously. In the second model, the groups with margins ≥ 1 mm were pooled to compare against the <1 mm group.

In both analyses, none of the tested variables emerged as a significant independent predictor for either local recurrence or OS. Detailed results are presented in Table 5.

## 4. Discussion

This study analyzed outcomes in a cohort of osteosarcoma patients treated at a tertiary sarcoma center, with a particular focus on the prognostic relevance of the soft tissue surgical margin width.

### 4.1. Demographic Data

Our cohort of 75 patients had a mean age of 22 years and a slight male predominance (53%), in line with epidemiological data indicating a peak incidence of osteosarcoma in adolescents and young adults [2,6]. In line with these epidemiological findings, the majority of tumors in our cohort were located in the lower extremities, with 51% in the femur and 24% in the tibia [2,4]. Approximately 19% of patients presented with metastases at diagnosis, and another 19% developed metastases during follow-up, consistent with previously reported rates in larger cohorts [7].

### 4.2. Local Recurrence

Univariate analysis identified three significant predictors of local recurrence: the presence or development of metastases, advanced UICC tumor stage, and pelvic tumor location. Patients with metastatic disease had a significantly higher risk of recurrence (*p* = 0.003), likely reflecting more aggressive tumor biology [13,26] or just coexistence in terms of synchronous metastatic spread. Tumor location was another highly significant factor (*p* < 0.001), with pelvic tumors resulting in more recurrences than extremity tumors. These results confirm earlier studies demonstrating that pelvic osteosarcomas are associated with poorer local control [14,27].

### 4.3. Overall Survival

In terms of OS, univariate analyses revealed that patients with metastatic disease (*p* = 0.002) and advanced tumor stages (*p* < 0.001) had significantly shorter survival times. This emphasizes the importance of early detection and aggressive treatment of localized disease, which is associated with better outcomes [7,8]. Our findings are consistent with the EURAMOS-1 study, which identified the presence of metastases at diagnosis as a major negative predictor of survival [26]. The significant impact of location (*p* = 0.001) on OS, with poorer outcomes for pelvic tumors, aligns with previous reports in the literature highlighting pelvic osteosarcomas as prognostically unfavorable [28].

The multivariate analysis did not identify significant factors for local recurrence or OS, likely reflecting the limited sample size and potential confounders.

### 4.4. Surgical Margins and Local Control

The significance of surgical margin width in osteosarcoma treatment remains debated. Our study found no significant difference in local recurrence when the three groups were compared overall (*p* = 0.074). However, when the very close resections (<1 mm) were compared to the rest of our cohort, a significant lower recurrence-free time was seen (*p* = 0.024). This could be a very important new finding, but does not contradict the previously published data.

Studies examining very close margins are lacking. Loh et al. reported bony margins between 50 and 15 mm [20], and Li et al. chose their cut-off at 5 mm [21], but neither reported higher recurrence rates in narrower margins. Andreou et al. and Bertrand et al. examined closer margins and compared patients receiving resections ≤1 mm and >1 mm. Andreou et al. observed the bony margins and Bertrand et al. observed soft tissue margins; in both cases, no significant difference in recurrence was seen [8,14]. In 2016, He et al. published a meta-analysis, which is the only paper so far to see a significant difference in recurrence between closer and wider resection margins; however, no accurate definition of these terms has been given, and they were probably different for each of the examined primary sources [13].

While the width of the surgical margin was the focus of our analysis, the qualitative nature of the resection margin—e.g., tumor adjacent to fascia, muscle, or perineurovascular sheaths—has recently gained attention as a potentially important prognostic factor [29]. Unfortunately, due to the retrospective design and the lack of standardized documentation in our pathology reports, we were unable to systematically assess margin quality in our cohort.

Our findings suggest that very close margins, although histologically negative, may carry an increased risk of recurrence—especially when tumors abut vital structures that limit resection. However, these findings must be interpreted cautiously, as the multivariate analysis did not confirm statistical significance. This may be due to the small sample size of group 1 (*n* = 8), limiting statistical power.

### 4.5. Surgical Margins and Overall Survival

Importantly, margin width was not associated with overall survival (*p* = 0.896), echoing prior studies that failed to show a survival difference between closer and wider resections [8,14,20,21]. This finding raises a critical clinical question: If very close margins increase the risk of recurrence but not mortality, should more aggressive surgical approaches, including amputations, be reconsidered in cases with close proximity to neurovascular structures?

Our data suggest that very close resections may be acceptable in anatomically constrained cases, as long as adequate follow-up and surgical salvage options are available. Local recurrence, while undesirable, may be managed with revision surgery without compromising long-term survival in many cases.

### 4.6. Limitations and Future Directions

This study has several limitations that must be considered. The retrospective design introduces the possibility of selection bias, and the relatively small sample size may have limited the power to detect significant differences in recurrence or survival rates. In particular, the very small number of resections with very close margins (group 1) poses a risk for statistical errors. Furthermore, osteosarcomas of different regions have been included due to the small sample size. As shown in our own cohort, pelvic tumors show worse outcomes than those of the extremities. However, they have been included in all groups and are therefore balancing each other out. Because histopathological data were lacking, we were unable to determine the quality of the surgical margin, such as whether the narrowest margin involved muscle tissue or was close to neurovascular structures.

Additionally, the inclusion of patients who underwent amputation may introduce a degree of heterogeneity, as these cases often represent more advanced tumor stages and differing biological behavior. However, we decided to retain amputations in the analysis, as histologically measurable soft tissue margins were documented in all cases, including narrow margins (<5 mm) in several amputations.

Even though local recurrence in osteosarcoma does not influence overall survival [26,30], it can have substantial clinical consequences for patients. As this was a retrospective study, we could not compare the impact of recurrent tumors on function and quality of life.

To evaluate the findings of this work, bigger, multicentric studies are needed. Furthermore, the impact of additional treatment, such as radiotherapy, should be evaluated, especially in patients receiving very close resections.

## 5. Conclusions

In contrast to previous studies, our results suggest that very narrow surgical margins may increase the risk of local recurrence, despite complete tumor resection. A larger-scale study will be necessary to clarify the relationship more clearly. Since recurrence does not appear to impact overall survival, limb-salvage surgery remains a justifiable approach—even with a higher risk of recurrence.

## Figures and Tables

**Table 1:** Patient demographic data and locations of the tumors for each of the three groups.

	Margin < 1 mm		Margin 1 to 5 mm		Margin > 5 mm		All	
Age	25		22		19		22	
Male	6	75%	20	49%	14	54%	40	53%
Female	2	25%	21	51%	12	46%	35	47%
Trunk	1	13%	3	7%	3	11%	7	10%
Upper Extremity	1	13%	6	15%	1	4%	8	11%
Lower Extremity	6	74%	32	78%	22	85%	60	79%
All	8	10%	41	55%	26	35%	75	

**Table 2:** Tumor characteristics for each of the three groups; size in mm. G: grading; T: extent of tumor; N: lymph node invasion; M: metastases; V: invasion of veins; L: invasion of lymphatic vessels; Pn: invasion of nerves.

		Margin < 1 mm		Margin 1 to 5 mm		Margin > 5 mm		All	
	Size	106		103		106		104	
Subentity	osteoblastic	3	38%	25	62%	15	57%	43	57%
	chondroblastic	0	0%	6	15%	7	27%	13	18%
	mixed	4	50%	4	10%	1	4%	9	13%
	telangiektatic	1	12%	5	13%	2	8%	8	11%
	high-grade surface	0	0%	0	0%	1	4%	1	1%
G	1	0	0%	0	0%	0	0%	0	0%
	2	0	0%	5	12%	2	8%	7	9%
	3	8	100%	36	88%	24	92%	68	91%
T	1	2	25%	14	34%	9	34%	25	33%
	2	6	75%	24	59%	15	58%	45	60%
	3	0	0%	1	2%	2	8%	3	4%
	4	0	0%	2	5%	0	0%	2	3%
N	0	0	0%	5	12%	10	38%	15	20%
	1	0	0%	0	0%	0	0%	0	0%
	x	8	100%	36	88%	16	62%	60	80%
M	0	3	37%	27	66%	17	65%	47	63%
	pre-surgery	1	13%	10	24%	3	12%	14	19%
	post-surgery	5	63%	14	34%	9	35%	28	37%
V	0	7	88%	39	95%	19	73%	65	87%
	1	1	12%	2	5%	7	27%	10	13%
L	0	8	100%	41	100%	26	100%	75	100%
	1	0	0%	0	0%	0	0%	0	0%
Pn	0	8	100%	41	100%	26	100%	75	100%
	1	0	0%	0	0%	0	0%	0	0%

**Table 3:** Pathological findings and treatment modalities for each of the three groups, margin width in mm.

		Margin < 1 mm		Margin 1 to 5 mm		Margin > 5 mm		All	
Margin width		0.5		2.0		28.2		20.2	
Grade of regression	1	0	0%	2	5%	0	0%	2	3%
	2	1	13%	7	17%	5	19%	13	17%
	3	1	13%	8	20%	7	27%	16	21%
	4	2	24%	18	44%	5	19%	25	33%
	5	4	50%	5	12%	7	27%	16	21%
	6	0	0%	1	2%	2	8%	3	5%
Surgical therapy	Endoprosthesis	6	74%	28	68%	9	35%	46	61%
	Biological reconstruction	1	13%	8	20%	1	4%	7	9%
	Amputation	0	0%	2	5%	12	46%	14	19%
	No reconstruction	1	13%	3	7%	4	15%	8	11%

**Table 4:** *p*-values for each of the variables used in univariate analyses concerning local recurrence and overall survival. Significant factors in bold.

	Recurrence	Overall Survival
Sex	0.855	0.550
Grading	0.497	0.574
Location	**<0.001**	**0.001**
Stage	**0.024**	**<0.001**
Response to chemotherapy	0.652	0.077
Metastases	**0.003**	**0.002**
Recurrence		0.482

**Table 5:** *p*-values for each of the variables used in multivariate analyses concerning local recurrence and overall survival.

	3 Groups		2 Groups	
	Recurrence	Overall Survival	Recurrence	Overall Survival
Sex	0.568	0.908	0.607	0.358
Age	0.977	0.982	0.640	0.679
Stage	0.202	0.918	0.070	0.916
Response to chemotherapy	0.406	0.964	0.640	0.492
Metastases	0.100	0.688	0.922	0.164
Location	0.313	0.994	0.562	0.156
Margin width	0.985	0.991	0.083	0.389
Size	0.166	0.989	0.210	0.674
Grading	0.085	0.914	0.908	0.908
Recurrence		0.926		0.310

## Data Availability

The original contributions presented in this study are included in the article. Further inquiries can be directed to the corresponding author(s).

## Abbreviations

The following abbreviations are used in this manuscript:

OS	Overall Survival
LR	Local Recurrence
LSS	Limb-Sparing Surgery
OS	Osteosarcoma
